# Novel and known *MYOC* exon 3 mutations in an admixed Peruvian primary open-angle glaucoma population

**Published:** 2012-08-08

**Authors:** Veronica Mendoza-Reinoso, Teja S. Patil, Maria L. Guevara-Fujita, Silvia Fernández, Enrique Vargas, Wilder Castillo-Herrera, Rodolfo Perez-Grossmann, Frank Lizaraso-Caparó, Julia E. Richards, Ricardo Fujita

**Affiliations:** 1 Centro de Genética y Biología Molecular (CGBM), Facultad de Medicina, Universidad de San Martín de Porres, Lima, Perú; 2 Department of Ophthalmology and Visual Sciences, W.K. Kellogg Eye Center, University of Michigan Medical School, Ann Arbor, MI; 3 Instituto de Oftalmología del Perú (INO), Lima, Perú; 4 Instituto de Glaucoma y Cataratas, Miraflores, Lima, Perú; 5 Department of Epidemiology, The University of Michigan School of Public Health, Ann Arbor, MI

## Abstract

**Purpose:**

The aim of this study was to characterize a representative sample of the Peruvian population suffering open-angle glaucoma (OAG) with respect to the myocilin gene (*MYOC*) mutations, glaucoma phenotype, and ancestry for future glaucoma risk assessment.

**Methods:**

DNA samples from 414 unrelated Peruvian subjects, including 205 open-angle glaucoma cases (10 juvenile glaucoma [JOAG], 19 normal-tension glaucoma [NTG], and 176 POAG) and 209 randomly sampled controls, were screened for nucleotide changes in *MYOC* exon 3 by conformational sensitive gel electrophoresis (CSGE) and mutation screening.

**Results:**

We identified a probable causative novel *MYOC* missense mutation, Gly326Ser, in one POAG case and found a consistent genotype-phenotype correlation in eight of his relatives. We also found the known causative *MYOC* mutation Trp286Arg in one JOAG case and one POAG case. A known causative single base *MYOC* deletion, T1357, was found in one POAG case. Two previously reported silent polymorphisms, Thr325Thr and Tyr347Tyr, were found in both the case and the control populations. A novel missense variant, Met476Arg, was identified in two unrelated controls.

**Conclusions:**

The screening of exon 3 of *MYOC* in a representative sample of 205 independent POAG patients from Peru and 209 matched controls identified novel and previously reported mutations (both pathogenic and nonpathogenic) from other global regions. These results reflect the complex admixture of Amerindian and Old World ancestry in urban populations of Latin America, in general, and in Peru, in particular. It will be important to gather information about the ancestral origin of *MYOC* and other POAG gene mutations to develop screening panels and risk assessment for POAG in Peru.

## Introduction

Primary open-angle glaucoma (POAG) is an optic neuropathy that causes visual impairment and irreversible blindness if undetected and untreated. Estimates worldwide indicate that it affects more than 45 million people and that it will affect 60 million by the year 2020 [1]. POAG has strong genetic components, as indicated by concordance in twin studies [2], linkage studies [3], and association studies [4]. There is also an increased risk of POAG among close relatives [5]. Additional evidence for genetic components of glaucoma comes from a biased disease frequency in populations of different ethnic origins, with greater risk of open-angle glaucoma (OAG) among people of African ancestry compared with those with European or Asian ancestry [5].

POAG is genetically complex and heterogeneous, with at least six causative loci, several regulatory genes and modes of inheritance, and different degrees of penetrance [5]. Among more than a dozen reported POAG loci [3], the first identified gene corresponded to the myocilin gene (*MYOC*), which is located in the GLC1A locus on chromosome 1q24. *MYOC* mutations are responsible for about 3% of late-onset POAG and a higher proportion of the earlier onset form called juvenile open-angle glaucoma (JOAG) [6,7]. Causative nonsense and missense *MYOC* mutations have been implicated in glaucoma in both families and populations, but other mutations that change the coding sequence are found in normal individuals [8-11]. POAG caused by *MYOC* mutations is inherited as an autosomal dominant disease in JOAG; however, in adults, the disease often shows incomplete penetrance [8,9]. The *MYOC* gene has three exons, and the third exon harbors most of the causal mutations described for this gene. This exon translates to an olfactomedin-like domain, which is highly conserved across species and is normally found in the extracellular matrix of the trabecular meshwork [12]. The normal activity of myocilin is not yet understood, but a gain-of-function mechanism for POAG pathogenesis is suggested by the mutation heterozygosity [12-14]. In addition the lack of pathology in absence of myocilin or in mutation homozygocity have been reported [15]. A recent study indicates that peroxisomal targeting of mutant myocilin appears to play a role in elevation of intraocular pressure [16].

There are different rare *MYOC* alleles and different frequencies of the more common *MYOC* alleles between different racial/ethnic groups around the world [10,13,15]. Most studies of POAG genetics, including studies of *MYOC*, have involved populations with Caucasian, African, and Asian ancestry [1,13,15]. Founder effects may be responsible for biased frequencies of specific *MYOC* alleles in different populations, such as more frequent occurrence of Gln368X and Arg480Lys in populations of European ancestry [13,17-21], Arg46X in mainland Asian populations, and Glu35Lys in populations of African ascent [22]. Thus, consideration of ancestry becomes an important issue when developing a profile of mutations in a population and undertaking a risk assessment of alleles in that population. With respect to Amerindians, we have reported the first POAG causal mutation of *MYOC*, 1440 C→A, in a family from the Southern Andean Peruvian region of Apurímac. Curiously, the mutation produces Arg480Lys, the amino acid shift reported to be the most common in Caucasian cases of POAG but produced by the transversion 1440 C→G [23].

South Americans are usually categorized as Hispanic or Latino, a terminology that encompasses a highly varied collection of individuals, with different degrees of admixture of native South American ancestry and European, African, and Asian ancestry. The genetic basis of diseases in this population has generally been poorly studied in comparison to other world populations.

Peru is a South American country with a multiethnic population that includes about 45% native South Americans, mainly from the Andes, but some from the Pacific coast and the Amazonian basin. The rest of the Peruvian population includes an admixed population (37% of Ameridian with Caucasians, Africans, and Asians), 15% Caucasian, 2% African Peruvian, and 1% Asian. Most of the population lives along the coast and in major cities in the Andes and the Amazon. In Peru, glaucoma is the second leading cause of blindness after cataracts. To understand the underlying causes of glaucoma and the risks accompanying different genotypes in the Peruvian population, we have been carrying out linkage and mutation screening in extended families [23-26].

In the present study, we used conformational sensitive gel electrophoresis (CSGE) [27], an indirect detection technique of single nucleotide variants, followed by sequencing to identify changes in the third exon of *MYOC*, to screen mutations in a sample of 205 Peruvian POAG cases and 209 controls. Identification of mutations in this admixed population allows us to begin cataloguing mutations relative to the different ethnic backgrounds from which they originate, which will assist with future risk-estimation studies in the genetically heterogeneous Peruvian population. These mutations will also increase our general knowledge about the correlation between mutations and phenotype.

## Methods

### Selection of cases and controls

The subjects were recruited according to a protocol approved by the Universidad de San Martín de Porres Institutional Review Board (IRB00003251-FWA0015320 valid until January 2013), and their informed consent was obtained. Two hundred and five POAG cases and 209 controls were included in this study. The heterogeneous ethnic background of the cases and the controls consists mainly of admixed individuals who combine Native South American, Caucasian, African, and Asian ancestry.

### Clinical examination

The ophthalmic examination included tests for visual acuity, analysis with a slit lamp, and gonioscopy. The Goldmann applanation tonometer was used to measure intraocular pressure (IOP), and optic nerve cupping was documented with a nonmydriatic camera. POAG inclusion criteria were the presence of glaucomatous changes of the optic disc, the open anterior-chamber angle, visual field loss, visual acuity, and the absence of secondary or developmental causes of glaucoma. The observation of a possible new causal mutation, (case G62), prompted us to extend the analysis to the patient’s sisters, nephews, and first cousins. Clinical examinations were performed at the Instituto de Oftalmología del Perú (INO), the Peruvian public national eye reference hospital (S.F. and E.V.), and the Instituto de Glaucoma y Cataratas (R.P-G. private service).

### Molecular analysis of MYOC mutations

Peripheral blood (3 to 5 ml) was collected from the cases and the controls in EDTA tubes (Vacutainer™; Becton Dickinson, Franklin Lakes, NJ). The samples were encoded for anonymity and kept under refrigeration and later transported to the Centro de Genética y Biología Molecular (CGBM) laboratory. DNA was extracted, and *MYOC* exon 3 was amplified using the polymerase chain reaction (PCR) with primers previously described by Shimizu et al. [10] and shown in Table 1. To verify the presence of mutations, the PCR products were analyzed using the CSGE technique adapted to our laboratory conditions [23]. Samples that showed distorted mobility on CSGE were cleaned with the QIAquick® PCR purification kit (QIAGEN, Germantown, MD) and sequenced at the DNA sequencing Core of the University of Michigan School of Medicine (Ann Arbor, MI) or Macrogen Inc. (Rockville, MD) using primers described previously for that purpose [10]. Sequence chromatograms were aligned to the reference sequence by the Basic Local Alignment Search Tool (blastn; National Center for Biotechnology Information, Bethesda, MD) and by Bioedit (Ibis Biosciences, Carlsbad, CA) and scored for mutations by visual inspection.

**Table 1:** Primer pairs 1F-2R and 3F-ST3R were used to amplify *MYOC* exon 3 in two segments to be screened by CSGE, and pair 1F-4R to amplify the entire exon for sequencing.

**Primer**	**Sequence**	**Annealing Temp. (°C)**	**Product length (bp)**
1F	5’-CTGGCTCTGCCAAGCTTCCGCATGA-3’	60	480
2R	5’-GAGGCCTGCTTCATCCACAGCCAAG-3’		
3F	5’-GAGCTGAATACCGAGACAGTGAAGGC-3’	56	586
ST3R	5’-GAAAGCAGTCAAAGCTGCC-3’		
1F	5’-CTGGCTCTGCCAAGCTTCCGCATGA-3’	60	967
4R	5’-GGCTGGCTCTCCCTTCAGCCTGCT-3’		

Sequencing was performed with the respective forward (F) and reverse (R) primer of the product detected mutated by CSGE.

## Results

### Sequence variants identified in the cases

The screening of *MYOC* exon 3 for mutations in 205 Peruvian OAG cases (including 10 JOAG and 19 NTG) and 209 age- and sex-matched controls revealed the presence of three causative mutations, (one novel), in four cases and three polymorphisms, (one novel), in 11 individuals. The clinical data and the sequence variants of the cases are shown in Table 2.

**Table 2:** *MYOC* exon 3 causative mutations found among 205 Peruvian POAG cases.

** **	** **	** **	** **	** **	**IOP (mm Hg)**		**Cup-to-disc ratio**	** **
**Mutation**	**Case**	**Gender**	**Ethnic origin**	**Age at diagnosis**	**RE**	**LE**	**RE/LE**	**Treatment**
Gly326Ser (976 G>A)	G62	Male	Afr And	50	30	30	0.9/0.8	Trabeculotomy/ Trabeculotoplasty
Trp286Arg (856 T→C)	G93	Female	And Cau	62	18	17	0.8/0.8	Glaucotensil
Trp286Arg (856 T→C)	G102	Male	And Cau	17	37	27	0.9/08	Vascular implant left eye. Panphotocoagulation
Tyr453MetfsX11 (1357delG)	G56	Female	Afr And	47	32	30	1/1	Isopto Carpine/ Trabeculotomy

#### 976 G→A (Gly326Ser)

**Figure 1:**
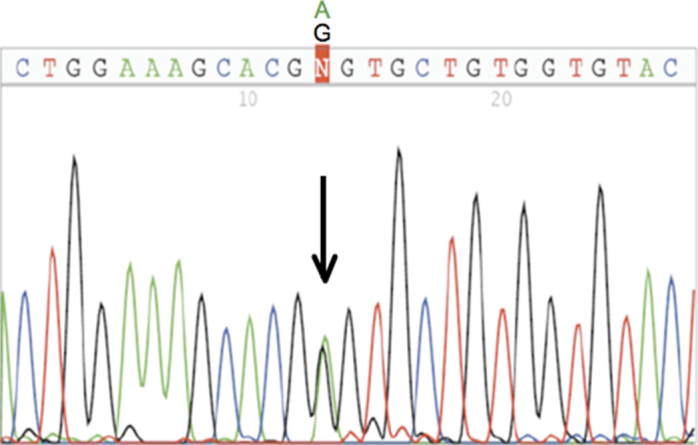
Novel mutation Gly326Ser caused by a transition G→A in nucleotide *MYOC* 976 changing codon GGT (Gly) to AGT (Ser). Found in case G62 and two of his first and second degree POAG affected relatives plus a younger relative with OHT.

The G to A transition at nucleotide 976 (Figure 1) caused a codon change, Gly326Ser, from a nonpolar to a neutral hydrophylic amino acid. This probable causative mutation was detected in case G62 (P), who was diagnosed with ocular hypertension (OHT; IOPs of 30 in both eyes) at age 59. At 65 years old (y.o.), pallor of the disc was detected, and at 69 y.o., the subject showed a cup-to-disc ratio of 0.9 in the right eye (RE) and 0.8 in the left eye (LE). He had trabeculoplasties and subsequently had trabeculotomy.

**Figure 2:**
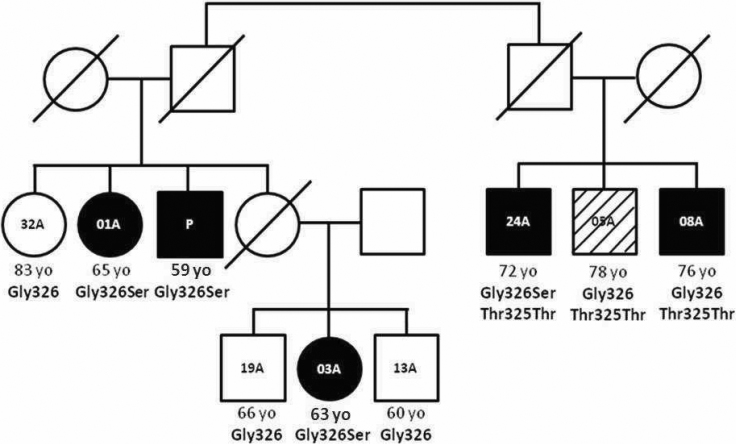
OHT and POAG family with *MYOC* Gly326Ser mutation and age of onset. The variant was detected in proband (P, case G62) and an extended analysis was performed in his closer relatives. Sister 01A with POAG, niece 03A has OHT with tendency to angle closure, and first cousin 24A with POAG are heterozygous for Gly326Ser. Cousin 05A has pseudoexfoliative glaucoma. Cousin 08A has been diagnosed with POAG. The three first cousins are heterozygous for *MYOC* Thr325Thr.

An extended analysis of his family with respect to the mutation (Figure 2, Table 3) demonstrated that an elder sister, 01A, carried the mutation. She was already blind, with terminal glaucoma identified at her first visit to the INO at 65 y.o., and she had total optic nerve excavation in both eyes. Another elder sister, 32A, last examined at 83 y.o., did not have POAG and was homozygous for normal Gly326. His niece, 03A, was heterozygous for Gly326Ser and diagnosed with OHT, with a tendency to angle closure at 63 y.o., and she had an iridotomy at 65. At 69 y.o., she presented with IOPs of 24 and 22 in the LE and the RE, respectively, despite timolol treatment. Two of her younger brothers, 13A (60 y.o.) and 19A (66 y.o.), do not have the mutation and do not have OHT or glaucoma yet. A first cousin, (24A), from the paternal side is heterozygous for Gly326Ser. At his first visit to the INO aged 72 y.o., he was diagnosed with terminal glaucoma, with a cup-to-disc ratio of 0.9 in both eyes and total disc pallor. He noted that he had been diagnosed with glaucoma in another hospital when he was 62 y.o. Two of his brothers do not have the Gly326Ser mutation but have glaucoma. One brother, 05A, at 78 y.o. presented with pseudoexfoliative glaucoma probably secondary to cardiovascular accident. The other brother, 08A, showed a later onset and milder presentation of glaucoma than his relatives with the mutation. He first visited the INO at age 76, with an IOP of 22 and 24 in the LE and RE respectively, optic nerve cupping of 0.6 (LE), and of 0.4 RE). After trabeculoplasty and treatment with thioctan and latanoprost case 08A conserved normal IOPs of 15 in both eyes up to his last check at age 82. These three first cousins are also heterozygous for polymorphism Thr325Thr. The proband, his nephews, and cousins were born in Cañete (Central coast) and have admixed African, Andean, and Caucasian ancestry.

**Table 3:** Cosegregation of heterozygous mutation Gly326Ser and OHT or POAG in the extended family of proband G62.

** **	** **	** **	** **	** **	**IOP**		**Visual acuity**		** **
**Patient code**	**Gender**	**Age Dx**	**Diagnosis**	**Gly326Ser**	**RE**	**LE**	**RE**	**LE**	**Surgical procedure**
G62	Male	59/65	OHT/POAG	yes	30	30	20/40	20/40	Trabeculectomy LE
01A	Female	65	Blind by POAG	yes	ptosis	20	HM	HM	-
03A	Female	63	OHT with tendency to angle closure	yes	26	20	20/30	20/30	Iridotomy
24A	Male	72	Advanced POAG	yes	26	26	HM	20/40	-
05A	Male	75	Pseudo exfoliation Glaucoma	no	25	17	-	20/40	Trabeculectomy, cataract surgery
08A	Male	76	POAG	no	22	23	20/50	20/40	Trabaculoplasty

#### Trp286Arg (856 T→C)

A transition in nucleotide 856 from T→C resulted in changes in codon 286 from a neutral nonpolar tryptophan to a positively charged arginine. Tryptophan at this position is very well conserved among mammals. Trp286Arg was previously found in one Caucasian POAG case and one Caucasian normal-tension glaucoma (NTG) case [13,28,29]. This missense mutation was detected in two independent cases. The first case, G93, was a mestizo female (Andean and Caucasian genetic background) from the Ica region (central coast). She reported previously visiting the regional hospital for high IOP. She presented to the INO aged 62 y.o. with a cup-to-disc ratio of 0.8 in both eyes. Subsequently she had been treated with dorzolamide, and the IOP was kept at 18 and 17 in the LE and RE, respectively. The second case, G102, an 18 y.o. male from the Lima Region (coastal province of Chancay) presented with JOAG with Trp286Arg with IOPs of 37 (RE) and 27 (LE) and a cup-to-disc ratio of 0.9 (RE) and 0.8 (LE). A vascular implant in the LE was performed in this case, and he was also treated with panphotocoagulation.

#### Tyr453MetfsX11 (1357delG)

A deletion of G at position 1357 was detected in case G56, a female of admixed African and Andean ancestry from Lima who was diagnosed with POAG when she was 47. She had IOPs of 32 (RE) and 30 (LE) and cup-to-disc ratios of 1.0 in each eye. This known mutation, common in individuals of African ancestry, is provoked by the deletion of guanine at nucleotide 1357. The molecular change causes a frameshift of the myocilin protein, starting at amino acid position 453, with a premature stop codon after 11 codons, resulting in a protein of 464 amino acids instead of the usual 504 [13,22].

### Sequence variants in six controls and five cases

The screening revealed three sequence variants, one novel and two previously reported benign polymorphisms, in six controls, as well as in five cases (Table 4).

**Table 4:** *MYOC* Exon 3 polymorphisms in 205 Peruvian POAG cases and 209 controls.

**Amino acid substitution**	**Base change**	**Fraction of cases (%)**	**Fraction of controls (%)**
Thr325Thr	975G→A	2/205 (0.97)	2/209 (0.96)
Tyr347Tyr	1041T→C	3/205 (1.46)	2/209 (0.96)
Met476Arg	1427T→G	0/205 (-)	2/209 (0.96)

#### Thr325Thr (975G→A)

A common synonymous polymorphism, Thr325Thr, resulting from a G→A transition at position 975, was found in two POAG cases and two controls. One case, from the coastal city of Chincha with the highest African ethnic input in Peru, was a 58 y.o. normal control with IOPs of 16 (RE) and 18 (LE) and a cup-to-disc ratio of 0.2 in both eyes. The other case, from the coastal city of Trujillo in the Northern La Libertad region, was a 57 y.o. control with IOPs of 12 (RE) and 16 (LE) and a cup-to-disc ratio of 0.2 in both eyes.

#### Tyr347Tyr (1041T→C)

Another previously reported *MYOC* polymorphism, Tyr347Tyr, resulting from a T→C transition at nucleotide position 1041, was found in three cases and two controls. All three cases had an admixed Amerindian and Caucasian background. One case came from the Amazonian city of Pucallpa, the second came from the Andean city of Cajamarca, and the third case came from the coastal region of Piura. Two controls, a 64 y.o. and a 77 y.o., also had this variant; they were both of admixed Andean and Caucasian backgrounds from the Andean cities of Ayacucho and Arequipa, respectively.

#### A novel polymorphism Met476Arg (1427T→G)

**Figure 3:**
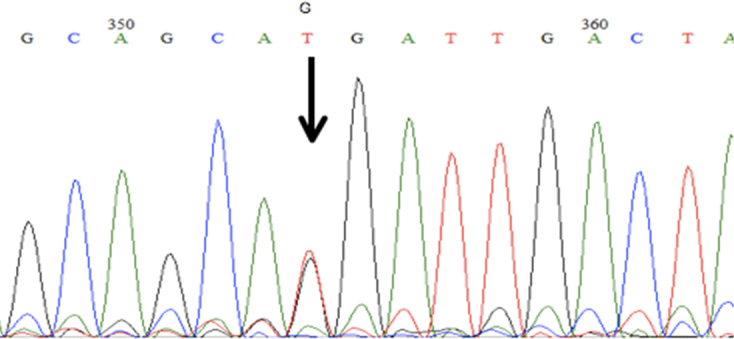
Novel polymorphism Met476Arg caused by a T→G transversion in nucleotide *MYOC* 1427 and the codon 476 from Met to Arg. This variant was found in 2 normal controls with neither glaucoma nor high IOP.

A novel transition from T to G at position 1427 (Figure 3) changed a conserved methionine at residue 476 to arginine, shifting the polarity from hydrophobic to hydrophilic. The accompanying charge change occurs in a region of the olfactomedin domain that has previously been reported to be apparently sensitive to charge changes [12,30]. This mutation was found in heterozygosity in two control individuals without glaucoma or high IOP and was absent in the cases. One of the controls with this variant (C124), was a 61 y.o. nonglaucomatous individual from Ancash (Central Andes) with IOPs of 15 (RE) and 13 (LE) and cup-to-disc ratios of 0.3 (RE) and 0.2 (LE). The other control (C199), with this variant was a 71 y.o. nonglaucomatous individual from Arequipa (Southern Andes) with IOPs of 20 (RE) and 14 (LE) and cup-to-disc ratios of 0.4 (RE) and 0.6 (LE).

## Discussion

Screening of *MYOC* exon 3 by CSGE in 205 OAG cases and 209 controls revealed two novel and four known sequence variants. Sequencing revealed two previously reported causal sequence variants, Trp286Arg (affecting two unrelated cases), and one case of a 1357delG deletion (affecting one case). A novel mutation, Gly326Ser (nucleotide 976, codon GGT to AGT), was also identified. The examination also showed two previously reported synonymous polymorphisms, Thr325Thr, (two cases and two controls), and Tyr347Tyr, (three cases and two controls). We also found another apparently previously unreported sequence variant, Met476Arg, in two independent controls (aged 61 and 71 y.o., respectively) with normal IOPs and cup-to-disc ratios.

### Novel *MYOC* Gly326Ser mutation probably causal of OHT and POAG

Sequencing revealed one novel sequence variant, Gly326Ser, in proband G62, with OHT detected at 59 y.o. and pallor of the disk at 65 y.o. An extended analysis of his relatives was undertaken to obtain a better insight into its causal involvement in POAG and the probable pattern of OHT early in the sixth decade and the development of glaucoma before the seventh decade. The mutation was present in his glaucomatous sister, who was declared blind at age 65; it was also in his niece, diagnosed with OHT with a tendency toward angle closure at 63 y.o. One first cousin, 24A, also had Gly326Ser and he had already been diagnosed with terminal glaucoma at age 72 at his first visit at INO; however, he had been diagnosed with POAG 10 years previously in another hospital. In contrast, Gly326Ser was absent in the proband’s older sister (unaffected at 83 y.o.) and in one of two apparently healthy nephews (brothers of affected niece 3A) last examined at 60 and 66 y.o. Neither had developed OHT or glaucoma yet. Although the two first cousins of G62 without the Gly326 variant had glaucoma, they had different phenotypes to the individuals with the Gly326Ser variant. One had pseudoexfoliative glaucoma probably due to a cardiovascular accident, and the other had POAG of a milder and older onset expression. This amino acid substitution, which changes nonpolar glycine to polar serine at position 326, is well conserved throughout mammalian evolution [30]. Further functional studies will be needed to determine categorically whether Gly326Ser is causal of OHT or POAG. However, the phenotype present in this family suggests that this mutation leads to OHT, which commences in the late 50s/early 60s and, if untreated, develops into glaucoma through the sixth decade.

### Ancestral origin of *MYOC* POAG causal mutations

Possible founder effects in the Peruvian population were seen in the POAG cases and the controls in the form of *MYOC* mutations previously identified in populations of European or African origin.

The novel mutation, Gly326Ser, was found in G62, a 72 y.o. male with an admixed African, Amerindian and Caucasian ancestry. The subject’s relatives bearing this mutation showed varying amounts of admixture of Amerindian, African, and Caucasian ancestries. Assignment of a probable ancestral origin of this variant is not yet possible.

The Trp286Arg POAG was reported previously as a causative mutation in cases of Caucasian ancestry in Europe and North America [12,13]. In our study, two of the 205 POAG cases showed this mutation. The mutation was present in an ethnically admixed (Andean and Caucasian) 69 y.o. POAG female, (G93), and in another mestizo (Andean and Caucasian) 18 y.o. diagnosed with JOAG. It is interesting to note that Trp286Arg has been reported in about one in every 700-1,000 (0.14%–0.1%) Caucasian POAG cases [13,28,29], whereas we found this variant in two of the 205 cases (0.97%). Whether this higher prevalence may be attributable to a stochastic event or to a founder effect would require an important increase of the sample of OAG cases. That is due to the low frequency of *MYOC* mutations in OAG (almost 2%) and the complex admixture of the Peruvian population.

The 1357delG mutation was found in one female POAG case from the coastal capital city of Lima. She has an African Andean admixed background and a family history of glaucoma. This mutation was previously detected only in African Americans [13] and in three South Africans of black African ancestry [22]. Thus, the most plausible origin of this POAG in Peru is a founder effect originating in African ancestry. During the colonial period, millions of sub-Saharan Africans were brought to the New World, including tens of thousands who were brought to Peru between 1521 and 1850. Presently, their descendents are an important contingent in the modern population of Peru, mainly in the coastal regions.

### Ancestral origin of *MYOC* polymorphisms in Peruvian cases and controls

For some *MYOC* sequence variants that have previously been seen in one specific population, determination of the ancestral origins can assist in interpreting the data, for example, the variant Thr325Thr 975G→A, a synonymous variant reported to be mainly from African ancestry and more rarely in Caucasians and Asians [10,12,13,22,31-37]. The four individuals detected in this study are from the coastal areas of Peru where African ancestors were more common, and one case and one control were both born in Chincha, the Peruvian city with the highest fraction of African ancestry. Thus, the most probable origin of this variant in these four individuals is from the African ancestry tracing back to colonial times, although origins from a different ancestral population cannot be ruled out when working with such an admixed population.

For some sequence variants that have been observed in many populations, use of ancestral information may not offer much assistance with data interpretation. The *MYOC* polymorphism, Tyr347Tyr, which we found in three cases and in two controls, is more often found in Caucasians, but it is also seen to a lesser extent in individuals with African, Indian, Japanese, and Chinese ancestry [12,13,38,39]. The five individuals with this sequence variant have an admixture of Caucasian and native (Coastal, Andean, or Amazonian) ancestry. Thus, the most likely origin of this variant in Peru is from Caucasian ancestry.

### Novel polymorphism MYOC Met476Arg is probably benign

The novel benign polymorphism 1427T→G (Met476Arg) resulted in a change of a nonpolar methionine at position 476, which is conserved across mammalian evolution, to a hydrophilic positively charged arginine. As it was found in two individuals aged 61 and 71 y.o. from the control (nonglaucomatous) group, both of whom had normal IOPs and cup-to-disc ratios, and it was not found in individuals with POAG, it seems more likely that this is not a causative mutation. At present we do not have any explanation for the innocuity of Met476Arg. Many of the charge changes in exon 3 are causative mutations, so there remains the possibility that the two unrelated controls with Met476Arg might go on to develop glaucoma later. On the other hand, in the screening of *MYOC* in different populations, several benign aminoacid mutations including changes of their polarity or their charge have been reported [9,13,22,28,29,36,38]. It has previously been reported that many causative changes occur in the olfactomedin domain of *MYOC* [12], raising the question of why the Gly326Ser mutation seems likely to be causative while the Met476Arg charge change variant seems to be a benign polymorphism. An answer can be the location relative to key sequences involved in amyloidogenesis [40]. The potentially causative charge change variant Gly326Ser is located two bases away from a core amyloidogenic section of the *MYOC* olfactomedin domain. The apparently benign Met476Arg variant is located near the COOH-terminal end in a region that has not been shown to play a role in amyloidogenesis [40]. The geographical origin of this variant mutation remains uncertain because both individuals were born in Andean cities where the most common ancestries are Andean and Caucasian. A study of single nucleotide polymorphisms and haplotypes that are representative of different ancestral populations would provide a better insight into the genetic background of this mutation.

In conclusion, the screening of exon 3 of *MYOC* in a representative cohort of 205 POAG individuals and 209 controls from the ethnically admixed population in Peru revealed four individuals with causative (1.95%) and 11 individuals with non causative mutations (2.42%). A novel mutation, Gly326Ser, studied in an extended family of mostly Amerindian and African ancestry showed probable causality for OHT and POAG with onset earlier to or before the sixth decade. The novel polymorphic variant Met476Arg was found in two normal controls, a 60 y.o. and a 70 y.o. with normal IOPs, with Andean and Caucasian genetic backgrounds. Further studies are necessary to determine Amerindian or Old World ancestry of these mutations. The known causative mutations Trp286Arg, and 1357delG were detected in cases of important Caucasian and African ethnic origin, respectively. The known polymorphic synonymous variants, Thr325Thr and Tyr347Tyr, were detected in individuals with African and Caucasian ancestry respectively. In these events the geographical origin of a previously reported Old World pathogenic or benign mutation corresponds quite well with the ethnic component of the Peruvian cases or controls. It is very likely that previously reported mutations correspond to a founder effect of Old World migrants in colonial Peru. The novel mutations require SNP haplotype analysis to determine their ethnic origin. For glaucoma risk assessment in complex admixed populations with native and migrant ancestries like the South American, in general, and the Peruvian, in particular, it is necessary to build a database of information from native and immigrant ancestry founder populations. Peruvian local geographic information can also assist in understanding differential ethnic risk factors for specific distributions. For example the coastal populations with greater African ancestry, urban populations with more Caucasian genetic background, or rural populations with more input of Native American ancestry.
